# Inhibiting HIF‐1α‐Induced miR‐663b Improves Pulmonary Arterial Hypertension by Reducing Dysfunctions of PASMCs Through Repressing Sirt1‐ZBP1‐Mediated PANoptosis

**DOI:** 10.1111/jcmm.71285

**Published:** 2026-08-03

**Authors:** Le Shen, Honghong Ma, Yang Yu, Yan Xu, Qun Yuan, Xia Zhao, Bing Zhuan

**Affiliations:** ^1^ Department of Respiratory Medicine People's Hospital of Ningxia Hui Autonomous Region Yinchuan Ningxia China; ^2^ Department of Respiratory Medicine, Third Clinical Medical College Ningxia Medical University Yinchuan Ningxia China; ^3^ Ningxia Medical University Yinchuan Ningxia China; ^4^ Suzhou Research Center of Medical School, Suzhou Hospital, Affiliated Hospital of Medical School Nanjing University Suzhou China

**Keywords:** AMPK, HIF‐1α, miR‐663b, PANoptosis, PASMCs, pulmonary artery hypertension

## Abstract

Pulmonary arterial hypertension (PAH) is a progressive disease characterised by vascular remodelling. Inflammatory responses and oxidative stress have been shown to contribute to its progression. This study investigated the effects of a novel HIF‐1α‐miR‐663b axis on pulmonary arterial smooth muscle inflammation, oxidative stress and apoptosis in a monocrotaline (MCT)‐induced PAH rat model. A PAH rat model was constructed via MCT injection; moreover, miR‐663b inhibitors were administered to inhibit miR‐663b. PASMCs were cultured under hypoxic conditions to establish an in vitro PAH model. The data revealed that miR‐663b was highly expressed in PAH rats and hypoxia‐induced PASMC models. Overexpression of miR‐663b exacerbated hypoxia‐induced proliferation, apoptosis, migration and inflammation in PASMCs. Conversely, miR‐663b inhibition reversed hypoxia‐induced dysfunction in PASMCs. ZBP1 downregulation dampened miR‐663b upregulation‐mediated dysfunctions in PASMCs. In vivo experiments revealed that miR‐663b inhibitors mitigated pulmonary arterial remodelling, inflammation and PANoptosis in MCT‐induced PAH rats. HIF‐1α expression was upregulated in hypoxia‐induced PASMCs. ChIP–qPCR and dual‐luciferase reporter gene assays confirmed that HIF‐1α transcriptionally regulated and promoted miR‐663b expression. Upregulation of miR‐663b inhibited AMPK/Sirt1 signalling pathway activation and promoted ZBP1‐mediated PANoptosis. However, miR‐663b inhibitors reversed hypoxia‐induced PANoptosis in PASMCs. The protective effects of miR‐663b inhibitors on PASMCs and their inhibitory effects on PANoptosis were significantly weakened by treatment with an AMPK inhibitor or a Sirt1 inhibitor. Collectively, these findings suggest that inhibiting HIF‐1α‐mediated miR‐663b expression improves PAH by reducing PASMC dysfunction through the attenuation of inflammatory responses, oxidative stress and apoptosis in PASMCs and the activation of the AMPK/Sirt1 pathway.

## 1 Introduction

Pulmonary arterial hypertension (PAH) is defined as a chronic, progressive disorder that is pathologically characterised by dyspnoea, reduced exercise tolerance and exertional syncope. The disease is characterised by heightened pulmonary vascular resistance and this abnormal increase sequentially results in right heart failure and fatal outcomes [1, 2]. The pathogenesis of PAH primarily involves sustained vasoconstriction, dysregulated proliferation and apoptosis of pulmonary artery smooth muscle cells (PASMCs) and endothelial dysfunction, ultimately leading to pulmonary vascular remodelling [3]. Studies have indicated that perivascular inflammation and oxidative stress are significant pathological characteristics of PAH [4, 5]. Currently, endothelin‐1, prostacyclin and nitric oxide are the three major therapeutic pathways. Although combining these pathways significantly improves the outcomes of PAH patients, long‐term survival is less acceptable [6]. Therefore, further investigations on its pathogenesis are necessary to identify novel therapeutic strategies against PAH.

MicroRNAs (miRNAs) constitute a class of small, endogenously expressed noncoding RNA molecules that are approximately 19–25 nucleotides in length. They are capable of regulating posttranscriptional gene expression profiles. miRNAs modulate various types of cellular functions, such as proliferation, differentiation, apoptosis and phenotypic transformation [7]. miRNAs are involved in the pathogenesis and progression of PAH. It has been reported that miR‐221 can facilitate the proliferation and migration of PASMCs in PAH [8]. Hypoxia treatment reduces the level of miR‐150 in rat lung tissues and plasma. Overexpressed miR‐150 inhibits the increase in right ventricular systolic pressure and reduction in cardiac output induced by hypoxia and lessens hypoxia‐driven thickening of the vascular wall [9]. miR‐663b is a member of the miRNA family and can promote the migration and tube formation of vascular endothelial cells [10]. Our research team reported that the expression of miR‐663b is increased in exosomes isolated from ‘M1’ macrophages and hypoxia‐induced PASMCs. Exosomal miR‐663b exacerbates hypoxia‐stimulated proliferation, inflammation, oxidative stress and the migration of PASMCs [11].

AMPK is an AMP‐dependent protein kinase that regulates a variety of catabolic and anabolic pathways. It maintains appropriate levels of ATP in cells in response to energy or cellular stress [12, 13]. AMPK acts not only as a cellular energy regulator but also as a sensor of redox signals. On the one hand, AMPK activation can reduce the production of superoxide; on the other hand, it can upregulate the expression of antioxidant genes to inhibit oxidative stress [14, 15]. AMPK activation attenuates hypoxia‐induced proliferation and resistance to apoptosis in PASMCs [16]. Sirt1 is an NAD^+^‐dependent deacetylase and a member of the Class III histone deacetylase (HDAC) family [17]. AMPK increases the level of NAD^+^ in cells to increase Sirt1 activity [18] and the activation of the latter can significantly ameliorate apoptosis, oxidative stress and inflammation [19]. Recent studies have suggested that activating the AMPK‐Sirt1 pathway can significantly reduce PASMC dysfunction in PAH models [20, 21].

Here, we reported that both miR‐663b expression and HIF‐1α expression were increased in a MCT‐induced PAH rat model. To further investigate the roles of HIF‐1α and miR‐663b in PAH, miR‐663b mimics and inhibitors were used to intervene in miR‐663b expression. We then evaluated alterations in the proliferation, inflammation, oxidative stress and apoptosis of PASMCs under hypoxia. The profiles of AMPK/Sirt1 pathway activity and HIF‐1α expression were also determined. We hope that this study provides a novel reference for PAH therapy.

## 2 Materials and Methods

### 2.1 Cell Culture and Hypoxia Induction

PASMCs were derived from the pulmonary arteries of male SD rats aged 5 weeks and were provided by the Experimental Animal Center of Ningxia Medical University Animal Center. As described previously [8], the animals were killed and their pulmonary arteries were separated and placed in cold PBS. After the adventitia and endothelium were removed, the pulmonary artery was severed into small pieces and kept in a cell culture flask filled with foetal bovine serum (10%), penicillin (100 U/mL) and streptomycin (100 U/mL). We cultured the flasks in a wet environment (5% CO_2_, 37°C). Immunostaining was performed with an anti‐α‐smooth muscle actin antibody (α‐SMA; Abcam, MA, USA) to identify primary cultured PASMCs. The third to seventh generations of cells were harvested for the following experiments. The cells in the normoxic group were exposed to 37°C, 21% O_2_, 5% CO_2_ and 74% N_2_ and those in the anoxic group were kept in a sealed chamber (Billups‐Rothberg) supplemented with 5% CO_2_ and 90%, 92% or 94% N_2_ to reach 5% O_2_, 3% O_2_ or 1% O_2._

### 2.2 Cell Transfection

Prior to transfection, PASMCs (density: 1 × 10^6^/mL) were maintained in DMEM supplemented with 0.1% FBS under anoxic conditions for 24 h. Lipofectamine 3000 reagent (Invitrogen, Carlsbad, CA, USA) served as the transfection tool for delivering HIF‐1α overexpression plasmids (HIF‐1α‐oe, 50 nM), small‐interfering RNA (siRNA) against ZBP1 (si‐ZBP1) and control vector or siRNA, miR‐663b mimics, the miR‐663b inhibitor and the corresponding negative control (50 nM each) following the manufacturer's instructions. PASMCs were treated with the HIF‐1α inhibitor LW6 (30 μM, InvivoChem), an AMPK inhibitor (compound C, 20 μM, #171261, Sigma‐Aldrich, Saint Louis, MO, USA) or a Sirt1 inhibitor (EX527, 40 μM, MedChemExpress, USA). Next, the transfected cells were exposed to an anoxic environment (3% O_2_, 5% CO_2_). After 48 h of transfection, the transfection efficiency was determined and the cells were used for subsequent experiments. Guangzhou RiboBio Co. Ltd. designed and synthesised all the plasmids.

### 2.3 RT–PCR

Total RNA from lung tissues or PASMCs was obtained using TRIzol reagent (Invitrogen, Carlsbad, CA, USA). DNAse‐I without RNase was used to treat the RNA samples for 20 min at room temperature (RT). As the purity was gauged, a Promega reverse transcription kit (Madison, WI, USA) was used to reverse transcribe the RNA and a TaqMan miRNA reverse transcription kit (Invitrogen) was used to reverse transcribe the mRNA. Amplification was performed with the assistance of an ABI PRISM 7300 fluorescent quantitative PCR system (Applied Biosystems, Foster City, CA, United States). The volume of the reaction mixture was 20 μL and the parameters for the reaction were set as follows: 95°C for 5 min, 95°C for 10 s, 60°C for 20 s and 72°C for 10 s. GAPDH and U6 were used as the internal parameters of HIF‐1α/ZBP1 and miR‐663b, respectively. The relative quantitative approach (2^−ΔΔ*C*T^) was used to determine the expression profiles of the target genes. The following specific primers were designed via the NCBI Primer‐Blast tool:

**Table:** 

Name	Forward (5′–3′)	Reverse (5′–3′)
HIF‐1α	AAGCACTAGACAAAGCTCACCTG	TTGACCATATCGCTGTCCAC
ZBP1	GTAGGTCAAAGGGTGGAGCC	GGCTGTGTGCTACTCCACAT
miR‐663b	CGCTAACAGTCTCCAGTC	GTGCAGGGTCCGAGGT
GAPDH	TGGTTGAGCACAGGGTACTT	CCAAGGAGTAAGACCCCTGG
U6	GCTTCGGCAGCACATATACT	GTGCAGGGTCCGAGGTATTC

### 2.4 Cell Counting Kit‐8 (CCK8)

PASMCs that were stably transfected and inoculated into 96‐well plates (density: 1 × 10^4^ cells/well) were precultured with 21% O_2_ and 5% CO_2_ at 37°C for 24 h. Following a 24‐h starvation period in medium containing 0.1% serum, the cells were incubated under normoxic or hypoxic conditions for 24, 48 or 72 h. Next, each well was given 10 μL of CCK8 solution (Beyotime Biotechnology, Shanghai, China) for another 4 h of culture. The absorbance (450 nm) was measured with a microplate reader (Bio‐Rad, CA, USA).

### 2.5 EdU Staining for Cell Proliferation

An EdU cell proliferation assay kit (CA1170; Solarbio Science & Technology) was used to evaluate the cell proliferation capacity. After 48 h of transfection, the PASMCs were incubated with EDU (50 μmol/L) culture medium for 2 h. The cells were then washed twice with PBS, fixed in 4% PFA for 30 min and incubated in 1× Apoll staining solution at 25°C under dark conditions for 30 min. The cell nuclei were stained with DAPI (D9542; Sigma‐Aldrich) and EdU‐positive cells were observed under a fluorescence microscope.

### 2.6 Transwell Migration Test

Transwell chambers (aperture 8 μm; Millipore, Billerica, MA, USA) were used to evaluate the migration of PASMCs. The cells were starved for 24 h. Next, PASMCs at a density of 5 × 10^4^ were suspended in SF medium (100 μL) and added to the upper compartment of the plate. The lower compartment was filled with 500 μL of medium supplemented with PDGF‐BB (catalogue number: HY‐P7087; MedChem Express). The cells were cultured at 37°C for 1 day. Cotton swabs were taken to gently wipe the upper chamber and the cells that migrated to the bottom were fixed with 3.7% PFA in PBS for 10 min and flushed with PBS for 5 min. After being dyed with 0.1% crystal violet (Sigma‐Aldrich, St. Louis, MO, USA) for 15 min, the cells were observed via an optical microscope for counting.

### 2.7 Western Blot

PASMCs were lysed in RIPA lysis buffer (Thermo Scientific, USA) supplemented with PMAF (1 mM). After the cell lysates were centrifuged, a BCA protein detection kit (Thermo Scientific, USA) was used to determine the protein concentration. Protein samples (30 μg) of comparable amounts were separated via 12% SDS–PAGE and transferred to PVDF membranes. The membranes were blocked using TBST supplemented with 5% skim milk powder for 2 h. Overnight incubation (4°C) of the membranes was performed using the following primary antibodies: anti‐ZBP‐1 (13285‐1‐AP, Proteintech, 1:500), anti‐NLRP3 (ab263899, Abcam, 1:1000), anti‐ASC (ab307560, Abcam, 1:1000), anti‐cleaved‐Casp1 (#4199, Cell Signalling Technology, 1:1000), anti‐GSDMD (ab219800, Abcam, 1:1000), anti‐Bax (ab32503, Abcam, 1:2000), anti‐cleaved‐Casp3 (ab214430, Abcam, 1:1000), anti‐cleaved‐Casp8 (#8592, Cell Signalling Technology, 1:1000), anti‐p‐RIPK1 (ab316923, Abcam, 1:1000), anti‐RIPK1 (ab300617, Abcam, 1:1000), anti‐p‐MLKL (ab196436, Abcam, 1:1000), anti‐MLKL (ab243142, Abcam,1:1000), anti‐p‐AMPK (ab133448, Abcam, 1:1000), anti‐AMPK (ab3759, Abcam,1:1500), anti‐Sirt1 (ab189494, Abcam, 1:2000), anti‐HIF‐1α (ab179483, Abcam, 1:2000) and anti‐GAPDH (ab181602, Abcam, 1:1000). The membranes were subsequently incubated with a goat anti‐rabbit secondary antibody (Ab6721; Abcam, 1:2000) conjugated with horseradish peroxidase (HRP) at RT for 1 h. An enhanced chemiluminescence detection kit (Thermo Scientific, USA) was used to visualise the bands. For normalisation purposes, β‐actin was employed as the internal reference.

### 2.8 ELISA

We harvested the cell supernatant and centrifuged it at 4°C and 1000*g* for 10 min to obtain the desired supernatant. As the rat lung tissues were collected, lysis buffer containing protease inhibitors (Sigma‐Aldrich) was used for homogenisation. The homogenate was centrifuged at 14,000*g* and 4°C for 25 min to produce the supernatant. As instructed by the supplier, the IL‐6, IL‐1β, IL‐18, TNF‐α, IL‐1α and IL‐33 levels in the cell and lung tissue supernatants were determined with ELISA kits (Abcam, Shanghai, China).

### 2.9 LDH Release Assay

The amount of LDH released from the PASMCs was measured via an LDH Cytotoxicity Assay Kit (Beyotime, Shanghai, China). Approximately 10^4^ PASMCs were incubated in 96‐well plates and cultured for 24 h. Following hypoxia induction, the PASMCs were incubated with LDH release reagent and centrifuged (400*g* for 5 min). Next, each well received 60 μL of LDH detection working solution, after which the plate was placed in an incubator at RT with protection from light. Thirty minutes later, the absorbance value at 490 nm was determined with a microplate reader. LDH activity was calculated as the fold change in the normoxia group.

### 2.10 Immunofluorescence and Immunohistochemistry

The lung tissue sections were first permeabilised with 0.1% Triton X‐100 for 30 min, followed by 1 h of 5% BSA blocking. The sections were then placed in incubation overnight at 4°C with a primary antibody against HIF‐1α (Abcam, ab179483), followed by overnight incubation with an Alexa Fluor488‐labelled secondary antibody (Abcam, ab150077). The cell nuclei were stained with DAPI solution (D9542; Sigma‐Aldrich) for 2 min and imaging was performed under a fluorescence Leica microscope. To determine α‐SMA expression in the lung sections, the prepared sections were incubated with anti‐α‐SMA antibody (1:200; #ab5694; Abcam) overnight at 4°C, followed by incubation with the secondary antibody. Finally, protein signal visualisation was achieved by diaminobenzidine (DAB) staining and haematoxylin counterstaining.

### 2.11 Construction of the Rat PAH Model

The Animal Center of the Experimental Animal Center of Ningxia Medical University was the provider of adult male SD rats (Approval number: SYXK (Ning) 2020‐0001). All schemes and operations were received from the Animal Care and Use Committee of the People's Hospital of Ningxia Hui Autonomous Region and were implemented as per the guidelines of the National Institutes of Health and the American Physiological Society. Proper measures were taken in the experiments to reduce the pain of the animals as much as possible. The rats were randomised to the sham group, PAH group, PAH + miR‐NC group or PAH + miR‐663b‐in group. MiR‐663b inhibitors were transfused into the caudal vein once a week. An 11‐membered macrocyclic pyrrolizidine alkaloid (PA), MCT, is extracted from the seeds of *Crotalaria spectabilis* . Among preclinical models of PAH, the MCT animal model has the advantages of mimicking several key aspects of human PAH, including vascular remodelling, the proliferation of smooth muscle cells, endothelial dysfunction, the upregulation of inflammatory cytokines and right ventricle failure and requires a single drug injection. To induce PAH, the rats received a single subcutaneous injection of MCT (50 mg/kg) [22]. All the animals were given unrestricted access to food and water while being kept in an environment with a 12‐h light/dark cycle at 25°C.

### 2.12 Hemodynamic Detection

Four weeks after MCT injection, the rats were weighed and anaesthetised via an intraperitoneal injection of ethyl carbamate (1 g/kg). The rats were fixed on the operating table and their right jugular veins were cut and exposed. A catheter linked to the biological function experimental system (Power Lab, AD Instruments, Australia) was inserted into the RV and we recorded the RVSP and the mean arterial pressure (mPAP). The animals were subsequently euthanised and subjected to thoracotomy. The RV, LV and interventricular septum (S) were dissected and weighed. The RV/(LV + S) ratio was used as an indicator of cardiac hypertrophy. The lung tissues were separated in preparation for subsequent trials.

### 2.13 HE Staining

The rats' lung tissues were immersed in 4% paraformaldehyde for fixation, after which they were embedded in paraffin and cut into 5 μm‐thick slices. Afterwards, they were subjected to routine HE staining. The morphology of the pulmonary arterioles was monitored with a light microscope. The vascular wall area percentage (WA%) for vessels with an outer diameter of 50–150 μm and the wall thickness percentage (WT%) were quantified using Image‐Pro Plus 5.1 software.

### 2.14 Chromatin Immunoprecipitation (ChIP)

A ChIP assay was conducted to test the binding affinity of HIF‐1α for the *miR‐663b* promoter using a ChIP Assay Kit (Cat/No. P2078, Beyotime, Shanghai, China). All procedures were conducted without the instructions of the producer. PASMCs were transfected with HIF‐1α‐oe or vector‐oe, as previously described. Twenty‐four hours after transfection, the cells were incubated with 1% formaldehyde at 37°C for 10 min. Afterwards, glycine solution was added to the cells at a final concentration of 125 mM. The cells were incubated at RT for 5 min. Next, the cells were collected and lysed with SDS lysis buffer (containing 1 mM PMSF). The DNA of the cells was fragmented into 200–800 bp segments by sonication. A 10% aliquot of each chromatin complex was reserved as an input control. IP was performed using anti‐HIF‐1α (ab179483; Abcam; 1:50) or rabbit IgG (1:50; Abcam) antibodies with Protein A/G Agarose/Salmon Sperm DNA to capture immune complexes associated with the miR‐663b promoter (site position: −1536 to −431). After sequential washes, the immunoprecipitated DNA was quantified using qPCR and the 2^−ΔΔ*C*t^ method [23].

### 2.15 Dual‐Luciferase Reporter Gene Assay

On the basis of predictions via JASPAR (http://jaspar.genereg.net/), the potential HIF‐1 binding sites on the promoter region of miR‐663b were identified. The promoter region (−1536 to −431) of miR‐663b was synthesised and inserted into the pRP[Pro]‐hRluc/Puro‐Luciferase reporter plasmids, which included a negative control, wild‐type (miR‐663b‐Wt) and mutant‐type (miR‐663b‐Mut). These plasmids were transfected into PASMCs. Twenty‐four hours after transfection, luciferase activity was evaluated using the Dual‐Luciferase Reporter Assay System (Promega) [24].

### 2.16 Statistical Analysis

Statistical analysis was performed with GraphPad Prism 8.0 software (GraphPad Software Inc., La Jolla, CA, USA). The outcomes are reported as the means ± standard deviations (SDs). Intergroup differences in statistical significance were analysed via Independent Student's *t*‐tests. Tukey's post hoc test, performed subsequent to one‐way ANOVA, was applied to analyse the differences between multiple groups. A *p* value less than 0.05 indicated statistical relevance.

## 3 Results

### 3.1 High Expression of miR‐663b in PAH Rat Models and Hypoxic PASMCs

**FIGURE 1:**
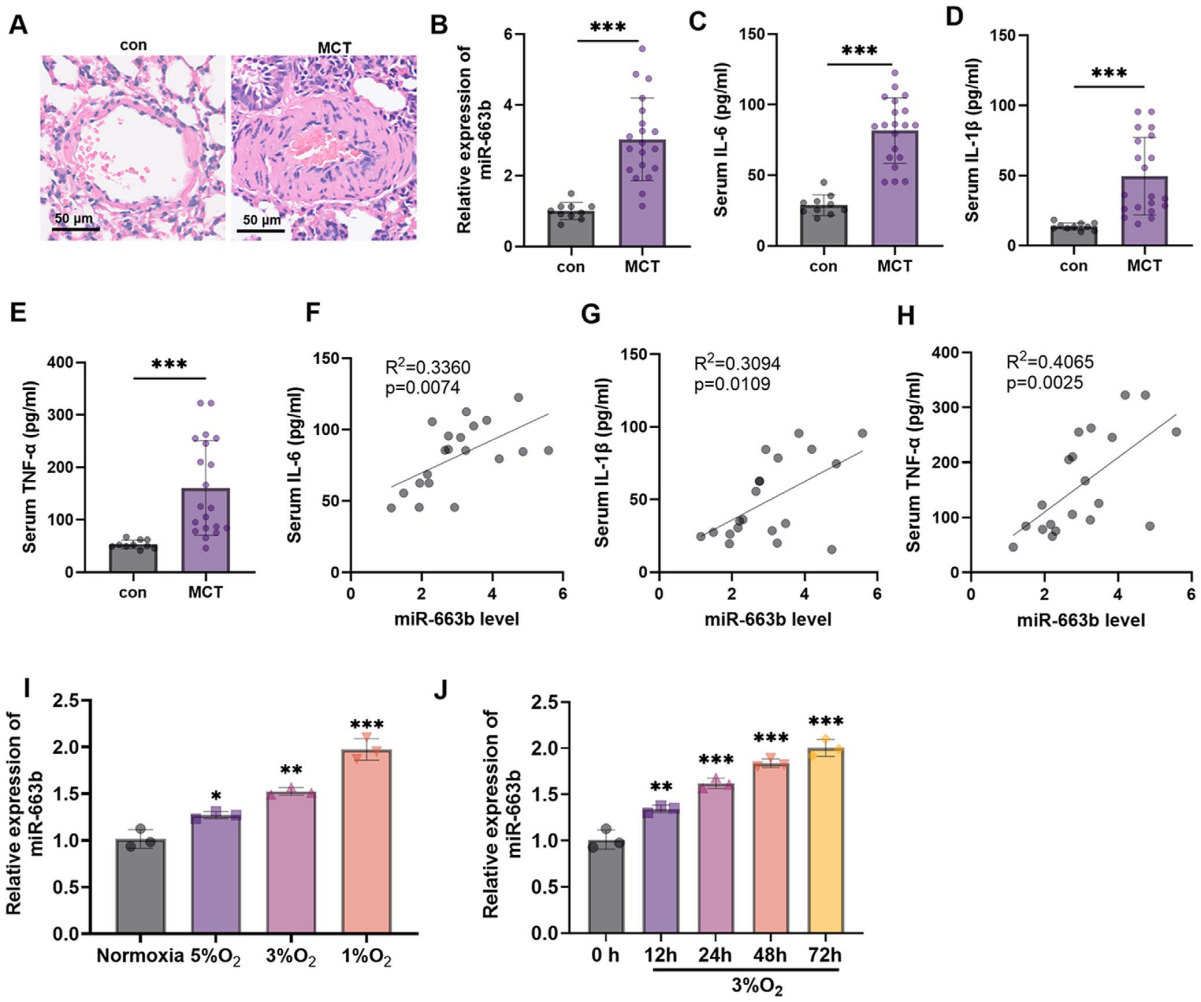
miR‐663b is highly expressed in rat PAH models and hypoxic PASMCs. An MCT‐induced PAH rat model was established. (A) H&E staining was used to observe histopathological changes in lung tissues. Scale bar = 50 μm. (B) RT–PCR analysis of miR‐663b expression in PAH model rats in the control group (*n* = 10) and MCT group (*n* = 20). ELISA quantification of the serum inflammatory markers IL‐6 (C), IL‐1β (D) and TNF‐α (E) in PAH rats (*n* = 20). Linear regression analysis was performed between the miR‐663b levels and the serum IL‐6 (F), IL‐1β (G) and TNF‐α (H) concentrations (*n* = 20). (I) Changes in miR‐663b expression in PASMCs under normal oxygen and 5%, 3% and 1% oxygen conditions (*n* = 3). (J) Changes in miR‐663b expression after different durations of exposure to 3% oxygen. *p < 0.05, **p < 0.01, ***p < 0.001 (two‐tailed unpaired Student's *t*‐test).

A PAH rat model was established by MCT administration. H&E staining revealed significant pulmonary artery remodelling (Figure 1A). We employed RT–PCR to detect miR‐663b expression in rat lung tissue. The data revealed that miR‐663b was markedly overexpressed in the lung tissue of MCT rats compared with that in the control group (*p* < 0.05; Figure 1B). ELISA kits were used to measure IL‐6, IL‐1β and TNF‐α levels in lung tissue homogenates and the results revealed significant increases in the levels of these cytokines in the MCT group (Figure 1C–E). Correlation analysis revealed that in the MCT group, the miR‐663b level was significantly positively correlated with the IL‐6, IL‐1β and TNF‐α levels (Figure 1F–H). The RT–PCR results revealed that miR‐663b expression significantly increased with decreasing oxygen concentration (21%, 5%, 3% and 1%) in PASMCs treated for 24 h (*p* < 0.05; Figure 1I). PASMCs were subjected to hypoxia (3% O_2_) for 12, 24, 48 or 72 h. RT–PCR analysis revealed a time‐dependent increase in miR‐663b expression (*p* < 0.05; Figure 1J). These results indicate that miR‐663b may facilitate the progression of PAH and modulate the function of PASMCs.

### 3.2 miR‐663b Enhanced the Proliferation and Migration of PASMCs Under Hypoxic Conditions

**FIGURE 2:**
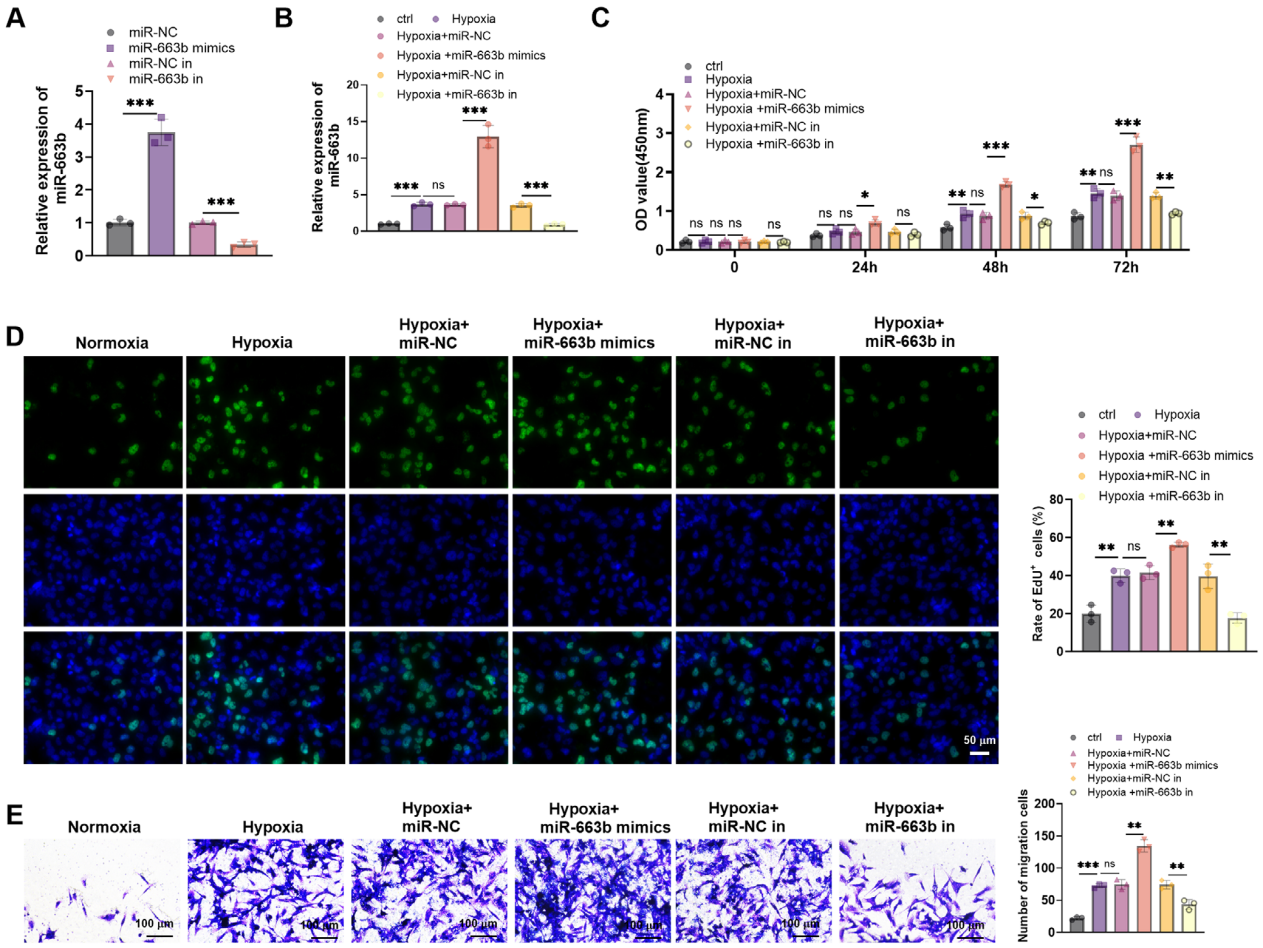
Effects of miR‐663b on hypoxia‐induced proliferation and migration of PASMCs. (A, B) MiR‐663b mimics and the miR‐663b inhibitor were transfected into PASMCs. PASMCs were cultured under normal oxygen or hypoxic conditions. The miR‐663b level was determined by RT–PCR. (C) A CCK‐8 assay was used to assess cell viability (*n* = 3). (D) Results of the EdU proliferation assay (*n* = 3). Scale bar: 50 μm. (E) Transwell assay for cell migration (*n* = 3). Scale bar = 100 μm. *p < 0.05, **p < 0.01, ***p < 0.001, ns *p* > 0.05, *N* = 3.

We transfected the miR‐663b mimics and miR‐663b inhibitors into the PASMCs (Figure 2A). The cells were then cultured under normoxic or hypoxic (3% O_2_) conditions. RT–PCR revealed that compared with the hypoxia + miR‐NC group, the miR‐663b mimic group had significantly higher miR‐663b levels, whereas the miR‐663b inhibitor group had lower miR‐663b levels (vs. the hypoxia + miR‐NC group) (Figure 2B). CCK8 and EdU assays demonstrated that hypoxia enhanced PASMC viability. The addition of miR‐663b mimics further increased PASMC viability, whereas the transfection of miR‐663b inhibitors suppressed this effect (Figure 2C,D). Transwell migration experiments revealed that compared with the hypoxia + miR‐NC group, the group in which miR‐663b mimics were added exhibited enhanced cell migration, whereas the group in which the miR‐663b inhibitor was added reversed the hypoxia‐induced migration of PASMCs (Figure 2E).

### 3.3 miR‐663b Promoted Inflammation and PANoptosis in PASMCs Under Hypoxic Conditions

**FIGURE 3:**
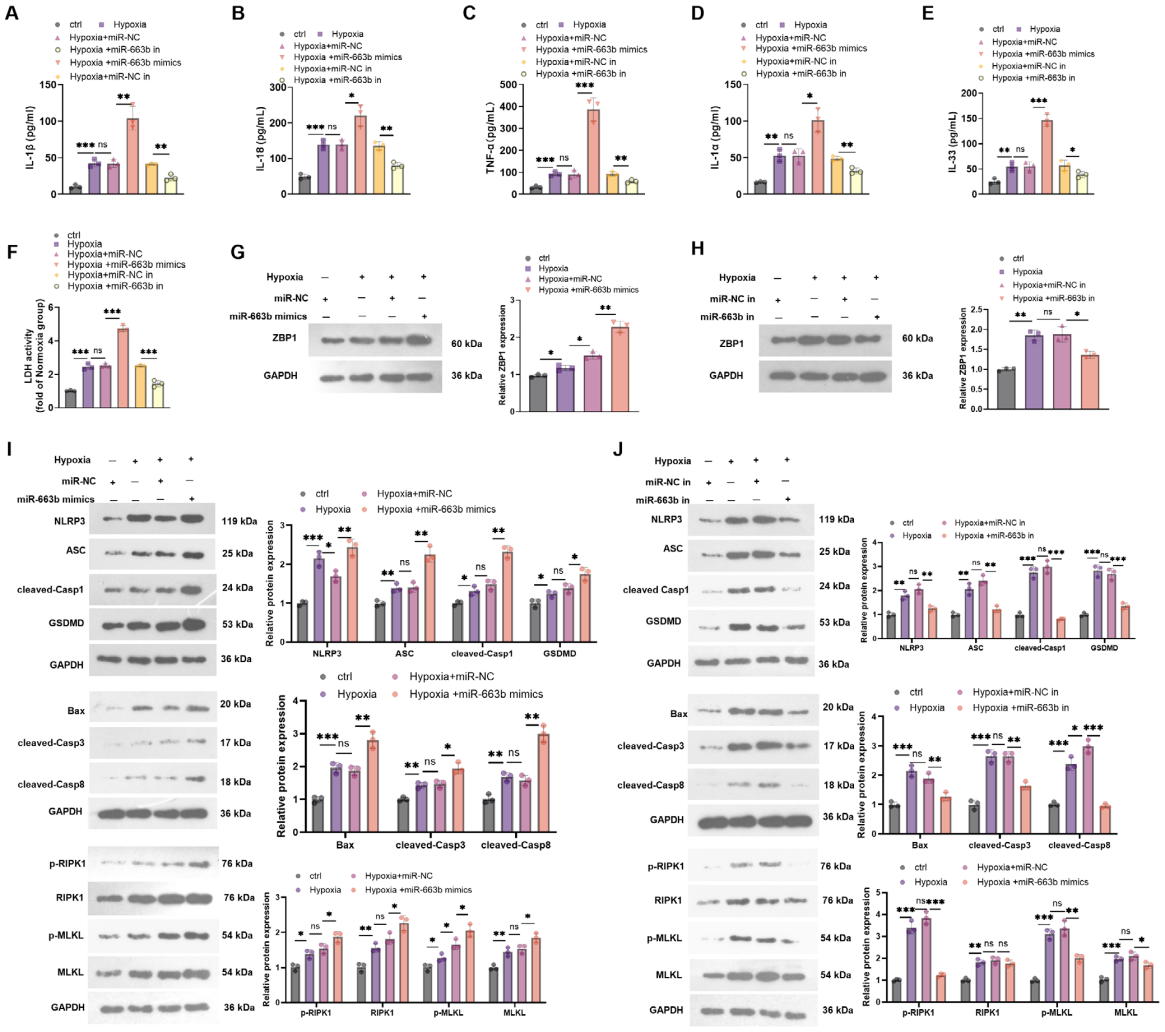
Effects of miR‐663b on hypoxia‐induced inflammation, apoptosis and necroptosis in PASMCs. miR‐663b mimics or inhibitors were transfected into PASMCs and the levels of intracellular inflammatory cytokines, including IL‐1β (A), IL‐18 (B), TNF‐α (C), IL‐1α (D) and IL‐33 (E), were measured via ELISA. (F) The release levels of intracellular LDH were assessed. (G–J) Western blot analysis revealed the expression of ZBP1, NLRP3, Casp1, GSDMD, Bax, Casp3, RIPK1 and MLKL1. *p < 0.05, **p < 0.01, ***p < 0.001; ns, *p* > 0.05 (two‐tailed unpaired Student's *t*‐test); *n* = 3.

ELISA was performed to determine the expression of inflammatory cytokines. The data revealed elevated levels of IL‐1β (Figure 3A), IL‐18 (Figure 3B), TNF‐α (Figure 3C), IL‐1α (Figure 3D) and IL‐33 in the supernatants of hypoxia‐treated cells compared with those in control cells. The miR‐663b mimic increased the expression of those cytokines, whereas the miR‐663b inhibitor reduced their expression (*p* < 0.05; Figure 3A–E). LDH release assays demonstrated that miR‐663b mimics amplified hypoxia‐induced LDH production, indicating that miR‐663b overexpression exacerbates hypoxia‐induced cellular damage. Treatment with miR‐663b inhibitors effectively reversed the hypoxia‐induced increase in LDH activity (*p* < 0.05; Figure 3F). We further investigated the expression of ZBP1 (the key ‘switch’ for PANoptosis), the pyroptosis executor protein GSDMD, the apoptosis executor protein caspase‐3/caspase‐8 and the necrosis executor protein MLKL. Western blot results demonstrated that treatment with miR‐663b mimics increased the expression of PANoptosis‐associated proteins, including ZBP1, NLRP3, cleaved Casp1, GSDMD, Bax, cleaved Casp3, cleaved Casp8, RIPK1 and MLKL. The miR‐663b inhibitor significantly suppressed hypoxia‐induced PANoptosis (Figure 3G–J). These findings confirm that miR‐663b inhibition inhibits hypoxia‐induced inflammatory responses and PANoptosis in PASMCs.

### 3.4 ZBP1 Downregulation Reversed miR‐663b‐Mediated Dysfunction and PANoptosis in PASMCs Under Hypoxic Conditions

**FIGURE 4:**
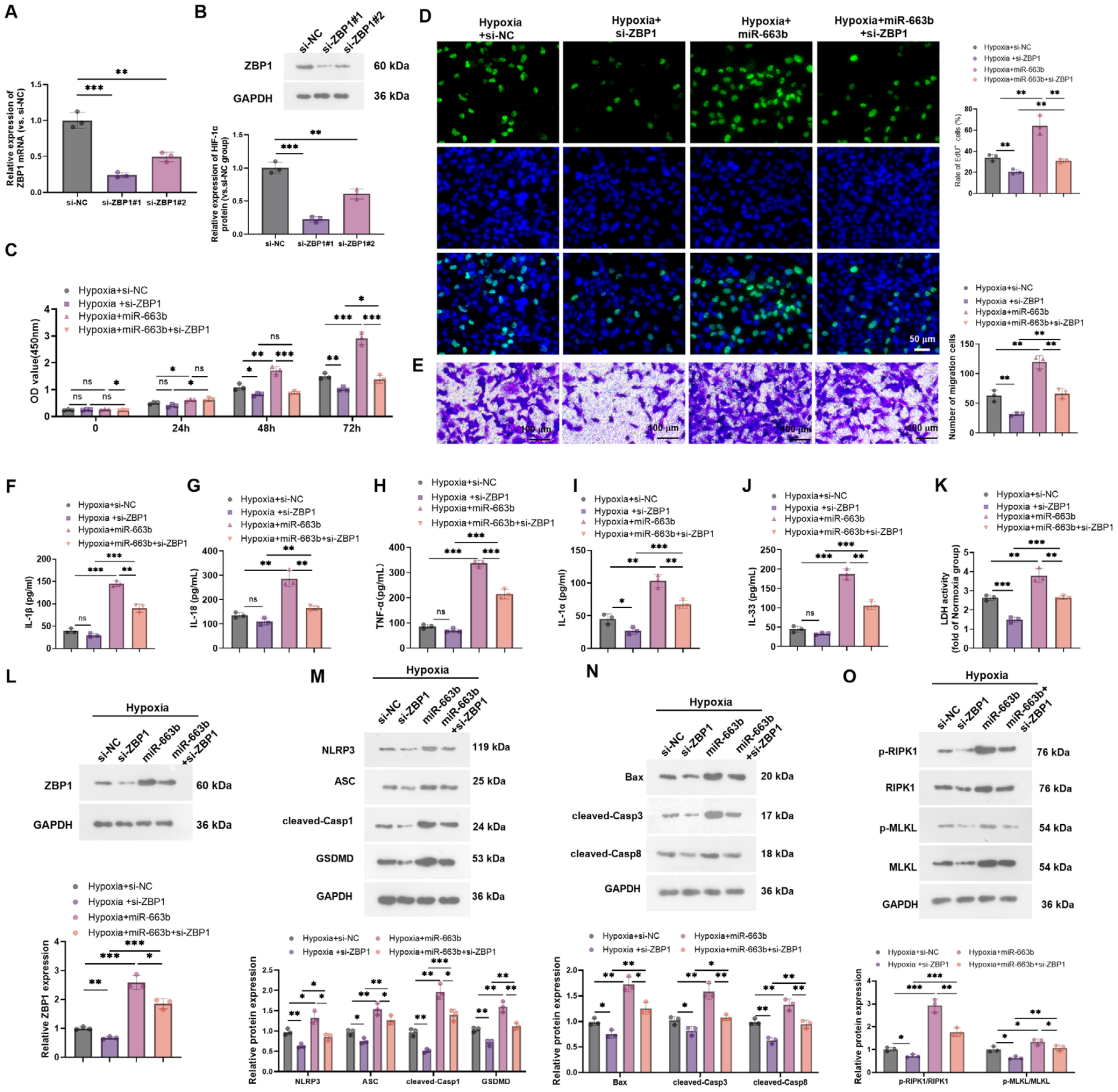
Effects of ZBP1 downregulation on miR‐663b‐mediated dysfunction in PASMCs under hypoxic conditions. (A, B) PASMCs were transfected with si‐NC or si‐ZBP1. RT–PCR and Western blotting were conducted to detect ZBP1 mRNA and protein levels, respectively. PASMCs were transfected with miR‐663b mimics and/or si‐ZBP1 and then cultured under hypoxic conditions. (C) A CCK‐8 assay was used to assess cell viability (*n* = 3). (D) Results of the EdU proliferation assay (*n* = 3). Scale bar: 50 μm. (E) Transwell assay for cell migration (*n* = 3). Scale bar = 100 μm. The levels of intracellular inflammatory cytokines, including IL‐1β (F), IL‐18 (G), TNF‐α (H), IL‐1α (I) and IL‐33 (J), were measured via ELISA. (K) The release levels of intracellular LDH were assessed. (L–O) Western blot analysis revealed the expression of ZBP1, NLRP3, Casp1, GSDMD, Bax, Casp3, RIPK1 and MLKL1. *p < 0.05, **p < 0.01, ***p < 0.001; ns, *p* > 0.05 (two‐tailed unpaired Student's *t*‐test); *n* = 3.

To verify the role of ZBP1 in the effects of miR‐663b, a ZBP1 downregulation model was constructed in PASMCs (Figure 4A,B). Functional assays were then performed. Compared with the hypoxia group, the ZBP1 downregulation group exhibited markedly reduced hypoxia‐induced proliferation and migration of PASMCs (Figure 4C–E). Notably, ZBP1 downregulation also partly reversed the miR‐663b‐induced proliferation and migration of PASMCs (vs. the hypoxia + miR‐663b group, Figure 4C–E). The cellular inflammatory and toxic reactions of PASMCs were determined. Compared with the Hypoxia + si‐NC group, the Hypoxia + si‐ZBP1 group had significantly reduced production of TNF‐α, IL‐1α and LDH (Figure 4F–K). The hypoxia+miR‐663b group had significantly elevated release of proinflammatory cytokines (including IL‐1β, IL‐18, TNF‐α, IL‐1α and IL‐33) and LDH. However, the downregulation of ZBP1 repressed all of those indicators (Figure 4F–K). Western blotting was then performed to test the expression of ZBP1 and PANoptosis‐related proteins in PASMCs. ZBP1 downregulation led to reduced expression of PANoptosis‐associated proteins, including ZBP1, NLRP3, cleaved Casp1, GSDMD, Bax, cleaved Casp3, cleaved Casp8, RIPK1 and MLKL, in PASMCs under hypoxic conditions. Although miR‐663b mimics promoted the expression of those proteins, the addition of si‐ZBP1 significantly suppressed PANoptosis (Figure 4L–O). These results indicated that ZBP1 downregulation inhibits miR‐663b‐mediated dysfunction and PANoptosis in PASMCs under hypoxic conditions.

### 3.5 Repressing miR‐663b Disturbed MCT‐Induced Pulmonary Artery Remodelling, Inflammation and Oxidative Stress in Rats

**FIGURE 5:**
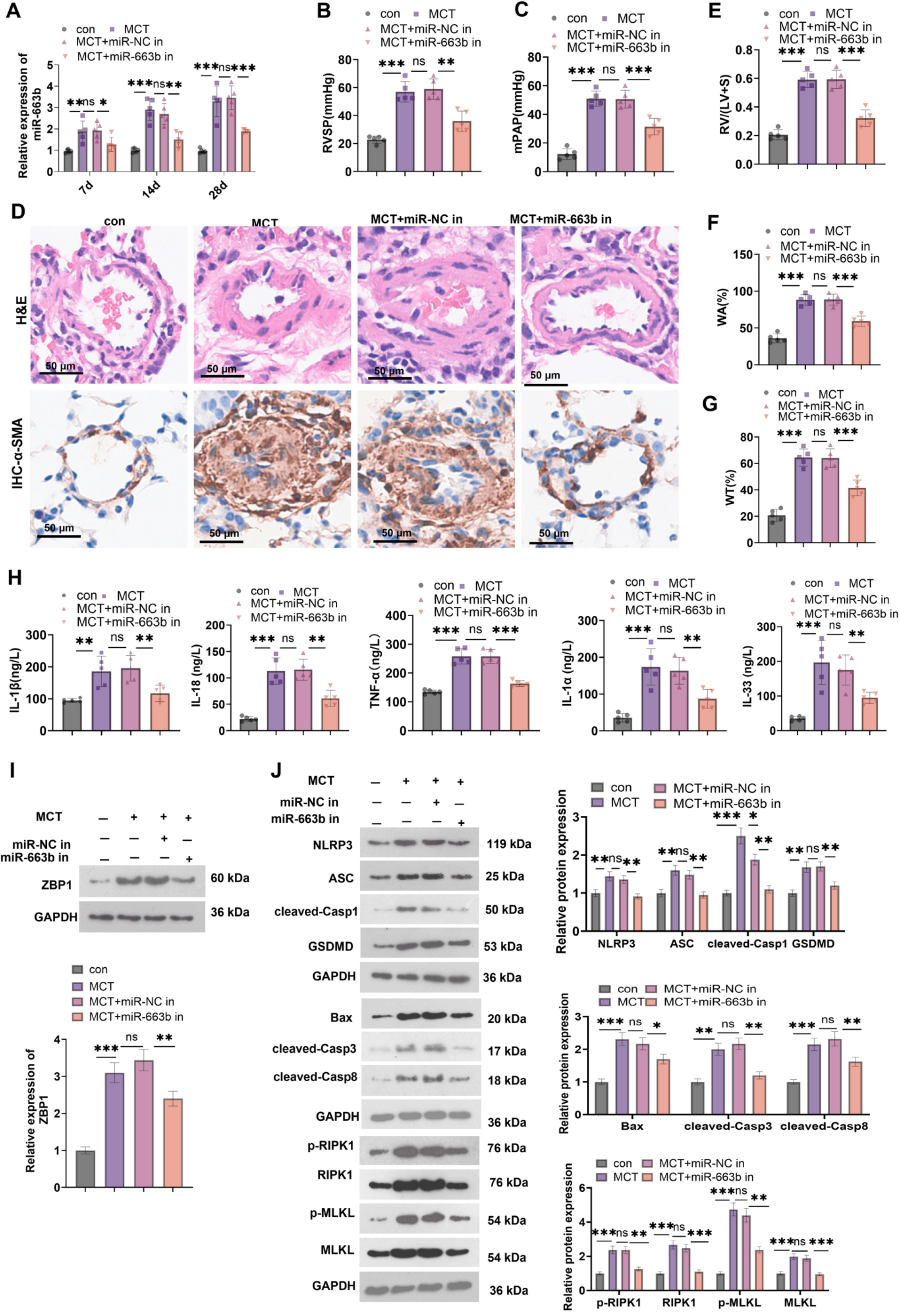
A miR‐663b inhibitor can inhibit hypoxia‐induced pulmonary artery remodelling, inflammation and oxidative stress in a PAH rat model. MiR‐663b inhibitors were administered via tail vein injection to rats, which were subsequently subjected to MCT injection for 28 days. (A) RT–PCR was used to detect miR‐663b expression in lung tissue. (B, C) RVSP and mPAP were measured via pressure sensors. (D) HE staining was performed to observe histopathological changes in the pulmonary vasculature. IHC was conducted to detect α‐SMA expression. Scale bar = 50 μm. (E) Rat heart RV/(LV + S). (F, G) Rat weight‐to‐body weight (WA) and weight‐to‐total body weight (WT%) analyses. (H) ELISA was used to quantify the serum levels of inflammatory cytokines (IL‐1β, IL‐18, TNF‐α, IL‐1α and IL‐33); *n* = 5. (I, J) Western blot analysis of the protein levels of ZBP1, NLRP3, Casp1, GSDMD, Bax, Casp3, RIPK1 and MLKL. *n* = 3. *p < 0.05, **p < 0.01, ***p < 0.001, ns *p* > 0.05 (two‐tailed unpaired Student's *t*‐test).

We established a PAH model in rats via intravenous injection of MCT and a miR‐663b inhibitor was administered weekly as a control. RT–PCR analysis of miR‐663b expression in PAH models revealed significant upregulation in rats injected with MCT, with marked downregulation observed in the presence of miR‐663b inhibitors (*p* < 0.05; Figure 5A). Compared with those in the con group, the levels of RVSP (Figure 5B) and mPAP (Figure 5C) were greater in the MCT group. Interference with the miR‐663b inhibitor reversed these effects. HE staining and IHC revealed thickened pulmonary arteries, narrowed lumens, inflammatory cell infiltration and increased α‐SMA expression in the MCT group. The miR‐663b inhibitor alleviated these pathological changes (Figure 5D). After the MCT treatment, the values of RV/(LV + S), WA (%) and WT (%) were significantly greater than those in the con group. Compared with the MCT + miR‐NC group, the miR‐663b inhibitor group had lower values for the aforementioned indices (Figure 5E–G). The levels of IL‐1β, IL‐18, IL‐6, TNF‐α, IL‐1α and IL‐33 in the lung tissues were detected via ELISA. These cytokines were significantly increased in the MCT group (vs. the con group). Treatment with the miR‐663b inhibitor reduced the expression of inflammatory factors (Figure 5H). Western blot analysis confirmed the expression of hallmark proteins in the pyroptosis, apoptosis and necrosis pathways. Compared with the control group, the MCT group exhibited increased expression of ZBP1, NLRP3, cleaved Casp1, GSDMD, Bax, cleaved Casp3, cleaved Casp8, p‐RIPK1 and p‐MLKL, while inhibition of miR‐663b markedly reduced their expression (Figure 5I,J). These findings demonstrate that miR‐663 inhibition alleviates pulmonary vascular remodelling, inflammation and PANoptosis in a rat PAH model.

### 3.6 HIF‐1α Enhances Hypoxia‐Induced Effects on PASMCs

**FIGURE 6:**
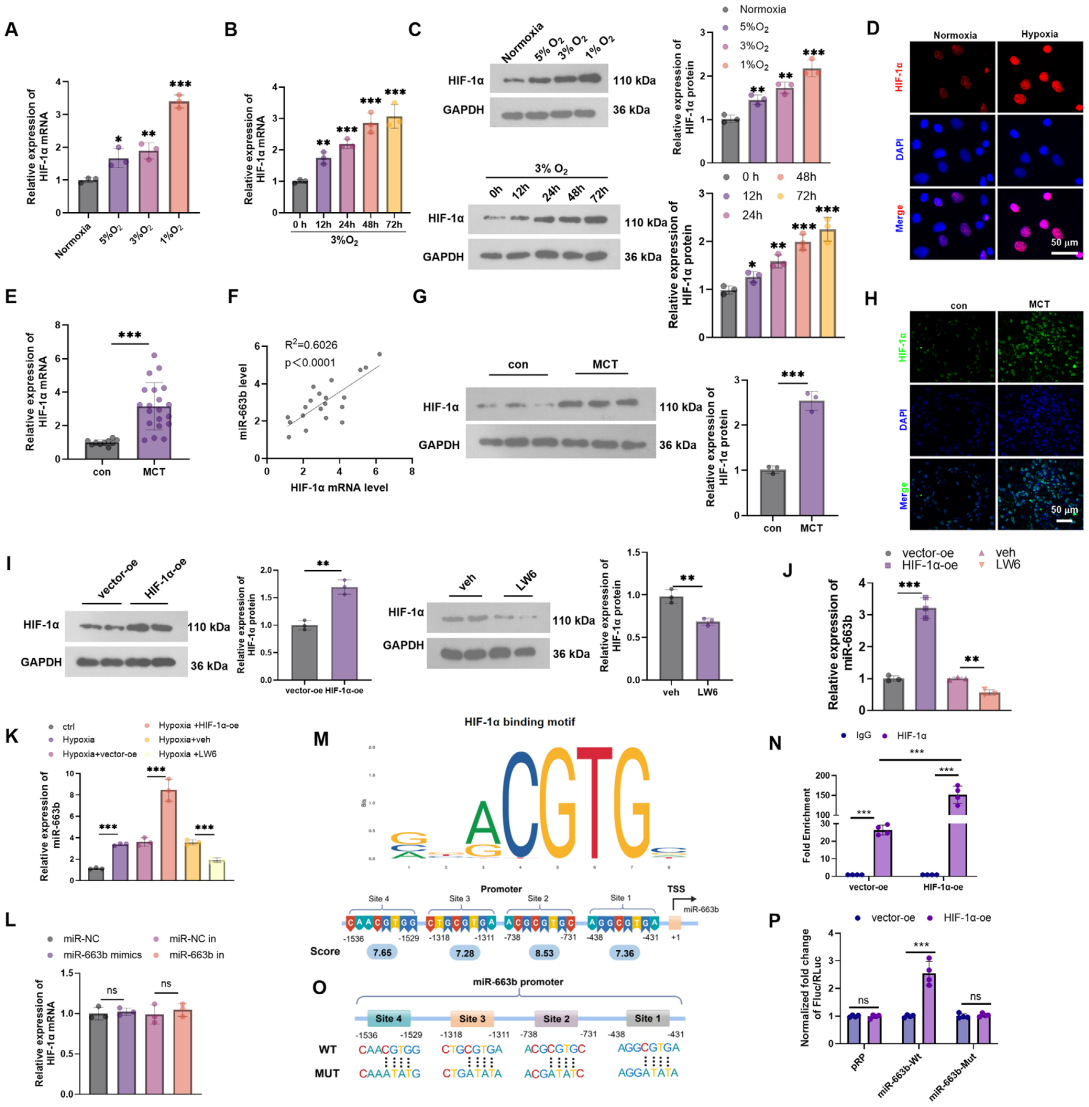
High expression of HIF‐1α in a rat PAH model and hypoxic PASMCs. (A) RT–PCR was used to detect changes in HIF‐1α expression in PASMCs under different oxygen concentrations (21%, 5%, 3% and 1%). (B) RT–PCR was used to detect changes in HIF‐1α expression in PASMCs under 3% oxygen for different durations. (C) Western blot analysis of HIF‐1α expression under various oxygen concentrations and exposure durations. (D) Immunofluorescence staining of HIF‐1α expression in cell nuclei under aerobic and hypoxic conditions. Scale bar: 50 μm. (E) RT–PCR analysis of HIF‐1α expression in the pulmonary tissues of PAH rats. (F) Linear regression analysis of miR‐663b levels versus HIF‐1α levels. Western blotting (G) and immunofluorescence staining (H) revealed HIF‐1α expression in PAH rat lungs. Western blotting (I) and RT–PCR (J) were used to assess the transfection efficiency of HIF‐1α‐oe. (K) RT–PCR was used to evaluate miR‐663b expression in PAMSCs with HIF‐1α‐oE or treated with LW6. (L) RT–PCR was used to measure the HIF‐1α mRNA level. (M) The potential HIF‐1α binding sites on the miR‐663b promoter were identified using JASPAR (http://jaspar.genereg.net/). (N) A ChIP–qPCR assay was conducted to test the interaction between HIF‐1α and the miR‐663b promoter (site position: −1536 to −431). (O) Dual‐luciferase reporter gene assay for the miR‐663b promoter. (P) Luciferase activity of the miR‐663b‐Wt/miR‐663b‐Mut promoter in the presence of vector‐oe or HIF‐1α‐oe. *p < 0.05, **p < 0.01, ***p < 0.001, ns *p* > 0.05 (two‐tailed unpaired Student's *t*‐test). *n* = 3.

We used RT–PCR and WB to analyse HIF‐1α expression in PASMCs under hypoxia. The results revealed that lower oxygen concentrations corresponded to higher HIF‐1α expression (Figure 6A–C), with HIF‐1α levels increasing in a time‐dependent manner under hypoxic conditions (Figure 6B,C). Immunofluorescence staining revealed that HIF‐1α levels were significantly greater in hypoxic cells than in normoxic cells (Figure 6D). Similarly, the expression level of HIF‐1α in the PAH model group was greater than that in the con group (*p* < 0.05; Figure 6E,G,H). Linear regression analysis revealed that the level of HIF‐1α was positively correlated with the level of miR‐663b (*R* ^2^ = 0.6026; Figure 6F). Next, we transfected HIF‐1α‐oe into PASMCs to upregulate HIF‐1α expression. The HIF‐1α inhibitor LW6 was added to the cells. Compared with that in the vector‐OE group, the expression level of HIF‐1α in the HIF‐1α‐oe group was significantly greater (*p* < 0.05). LW6 treatment significantly attenuated HIF‐1α expression (Figure 6I). When HIF‐1α was overexpressed, the expression level of miR‐663b was significantly upregulated. In contrast, the HIF‐1α inhibitor LW6 significantly reduced the level of miR‐663b (Figure 6J,K). Notably, when we inhibited the level of miR‐663b, the mRNA level of HIF‐1α did not significantly change (Figure 6L). The JASPAR database indicated that HIF‐1α can bind directly to the promoter of miR‐663b (Figure 6M). We subsequently performed ChIP–qPCR and the results indicated robust binding affinity of HIF‐1α to the miR‐663b promoter (Figure 6N). The promoter region (−1536 to −431) of miR‐663b was synthesised and inserted into pRP[Pro]‐hRluc/Puro‐luciferase reporter plasmids, which included negative control, wild‐type (miR‐663b‐Wt) and mutant‐type (miR‐663b‐Mut) plasmids, which were subsequently transfected into PASMCs (Figure 6O). A dual‐luciferase reporter gene assay indicated that HIF‐1α enhanced the activity of the miR‐663b‐Wt promoter but had no significant effect on that of the miR‐663b‐Mut promoter (Figure 6P). These results collectively indicated that HIF‐1α promoted the transcription of miR‐663b.

### 3.7 The Effect of HIF‐1α Targeting of miR‐663b on Hypoxia‐Induced PASMCs

**FIGURE 7:**
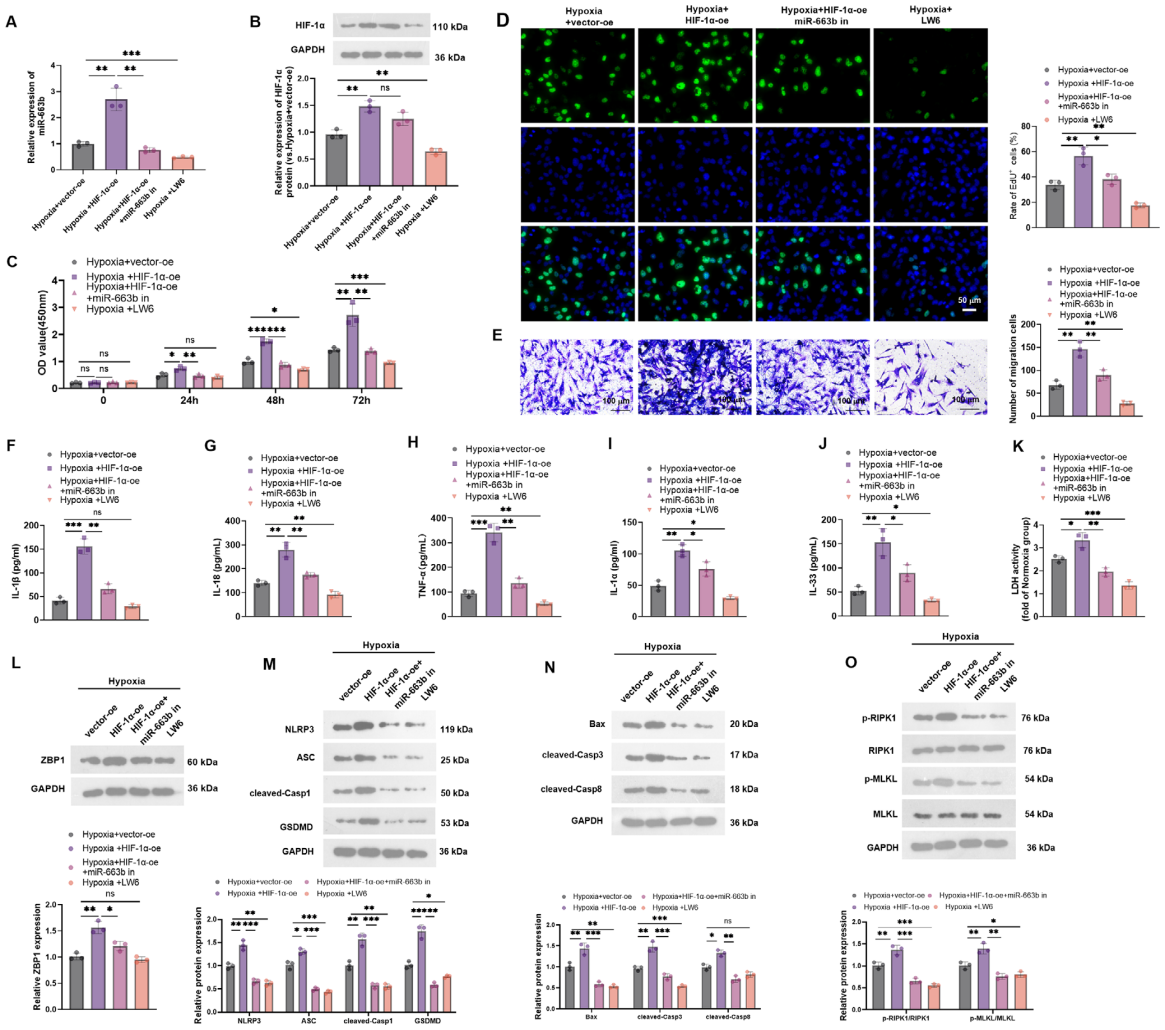
miR‐663b inhibition reversed HIF‐1α‐induced proliferation, migration, inflammation and PANoptosis of PASMCs. MiR‐663b‐inhibitors or HIF‐1α‐oe were transfected into PASMCs. LW6 was used to inhibit HIF‐1α expression. The cells were subsequently cultured under hypoxic conditions (3% O_2_). (A) RT–PCR was used to detect the effects of HIF‐1α‐oe or LW6 on miR‐663b levels. (B) Western blotting was used to measure HIF‐1α protein levels. (C) A CCK8 assay was used to detect cell viability. (D) EdU was used to detect cell proliferation. Scale bar: 50 μm. (E) Transwell assays were used to detect cell migration. Scale bar: 100 μm. (F–J) ELISA was used to analyse the levels of inflammatory cytokines (IL‐1β, IL‐18, TNF‐α, IL‐1α and IL‐33) in the supernatants of hypoxia‐treated cells. (K) LDH levels were measured. (L–O) Western blotting was used to measure the expression of ZBP1, NLRP3, Casp1, GSDMD, Bax, Casp3, RIPK1 and MLKL. *p < 0.05, **p < 0.01, ***p < 0.001, ns *p* > 0.05 (two‐tailed unpaired Student's *t*‐test). *n* = 3.

To further confirm the regulatory role of the HIF‐1α‐miR‐663b axis in hypoxia‐induced PASMCs, miR‐663b inhibitors, HIF‐1α overexpression plasmids and the HIF‐1α inhibitor LW6 were separately administered to hypoxic PASMCs. The results demonstrated that the overexpression of HIF‐1α enhances miR‐663b expression, whereas the suppression of HIF‐1α directly reduces miR‐663b expression, further confirming the regulatory role of HIF‐1α upstream of miR‐663b (Figure 7A). When miR‐663b expression was suppressed, HIF‐1α protein levels remained unchanged, indicating that miR‐663b does not regulate HIF‐1α expression (Figure 7B). Furthermore, we observed that HIF‐1α upregulation increased the proliferative activity and migration capacity of PASMCs. These alterations could be effectively alleviated by inhibiting miR‐663b or HIF‐1α (Figure 7C–E). We also examined the changes in inflammatory cytokines and PANoptosis markers. HIF‐1α overexpression increased the levels of inflammatory factors, including IL‐1β (Figure 7F), IL‐18 (Figure 7G), TNF‐α (Figure 7H), IL‐1α (Figure 7I), IL‐33 (Figure 7J) and LDH (Figure 7K) and the levels of proteins involved in pyroptosis, apoptosis and necrosis (Figure 7L–O). However, miR‐663b inhibition reversed HIF‐1α‐mediated effects. Moreover, LW6 treatment effectively reduced hypoxia‐induced inflammation and PANoptosis in PASMCs (vs. the hypoxia group; Figure 7L–O). These findings suggest that HIF‐1α may target miR‐663b to regulate inflammatory factors and the PANoptosis pathway.

### 3.8 Inhibiting AMPK/Sirt1 Signalling Diminished the Protective Effect of the miR‐663b Inhibitor on Hypoxic PASMCs

**FIGURE 8:**
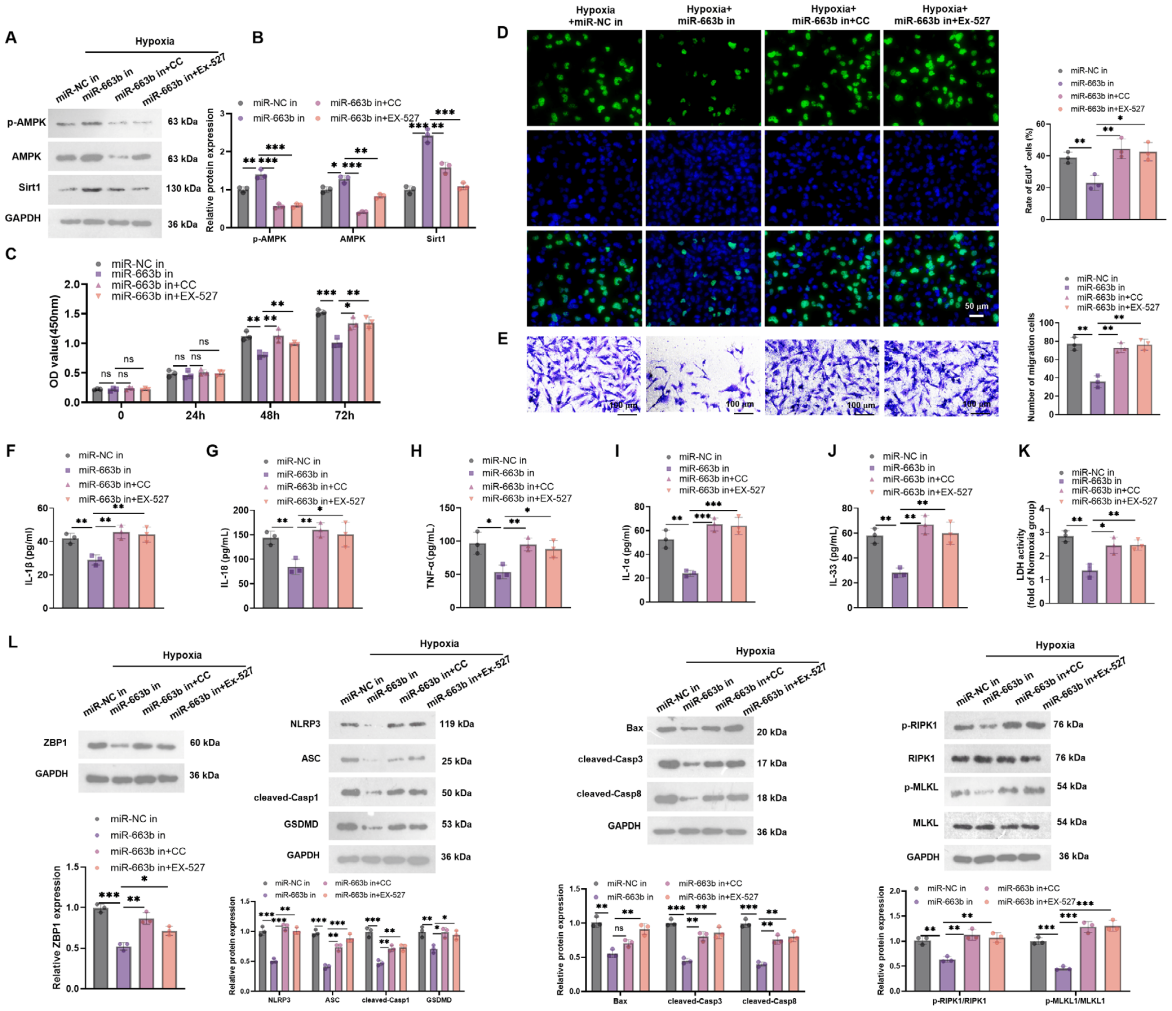
Inhibition of the AMPK/Sirt1 pathway attenuates the protective effect of the miR‐663b inhibitor on hypoxic PASMCs. PASMCs were transfected with miR‐663b inhibitors and treated with or without the AMPK inhibitor CC or the Sirt1 inhibitor EX‐527. (A, B) Western blot for detecting the expression of AMPK/Sirt1 in hypoxic PASMCs. (C) CCK8 assay for cell viability. (D) EdU assay for cell proliferation. Scale bar: 50 μm. (E) Transwell assay for cell migration; scale bar: 100 μm. (F, J) ELISA analysis of inflammatory cytokines (IL‐1β, IL‐18, TNF‐α, IL‐1α and IL‐33) in the supernatants of hypoxia‐treated cells. (K) Measurement of LDH levels. (L) Western blot analysis of ZBP1, NLRP3, Casp1, GSDMD, Bax, Casp3, RIPK1 and MLKL expression. *p < 0.05, **p < 0.01, ***p < 0.001, ns *p* > 0.05 (two‐tailed unpaired Student's *t*‐test). *n* = 3.

We further investigated the interaction between miR‐663b and the downstream AMPK/Sirt1 pathway to elucidate their relationship. miR‐663b inhibitors and their negative controls were transfected into PASMCs, after which the AMPK inhibitor Compound C and the Sirt1 inhibitor Ex‐527 were added and the cells were cultured under hypoxic conditions (3% O_2_). The western blot results indicate that inhibiting miR‐663b enhances AMPK phosphorylation and Sirt1 expression. When compound C was administered, AMPK phosphorylation and Sirt1 protein levels were reduced. Similarly, Ex‐527 synergistically reduced AMPK phosphorylation and Sirt1 expression (Figure 8A,B). CCK8, EdU and Transwell assays were used to evaluate cell viability, proliferation and migration. Compared with the Hypoxia + miR‐663b‐in group, both AMPK and Sirt1 inhibition enhanced these parameters (Figure 8C–E). ELISA and LDH assays revealed that AMPK and Sirt1 inhibition increased the levels of inflammatory mediators, including IL‐1β, IL‐18, TNF‐α, IL‐1α and IL‐33 and increased LDH levels (Figure 8F–K). Moreover, inhibition of AMPK and Sirt1 expression increased the expression of ZBP1, NLRP3, cleaved Casp1, GSDMD, Bax, cleaved Casp3, cleaved Casp8, p‐RIPK1 and p‐MLKL (Figure 8L). These data suggested that inhibiting the AMPK/Sirt1 pathway could counteract the protective effect of the miR‐663b inhibitor on hypoxic PASMCs.

## 4 Discussion

Altered miRNAs have been identified as hallmarks of PAH [25]. Targeting miRNAs has therapeutic effects on PAH [26]. The results of this study revealed that the expression of miR‐663b was significantly upregulated in the PAH rat model and in hypoxia‐induced PASMCs, indicating that miR‐663b serves as a marker for the pathogenesis of PAH. Functional experiments confirmed that inhibiting miR‐663b could target and improve the migration of PASMCs and reduce the release of inflammatory cytokines. Inhibiting miR‐663b alleviated pulmonary artery remodelling and reduced the right ventricular pressure load. Moreover, HIF‐1α, acting as an upstream regulatory factor, can directly activate the expression of miR‐663b, thereby inducing PASMC dysfunction. This study provides a novel HIF‐1α‐miR‐663b axis for the treatment of PAH.

PAH is a rare dyspnea–fatigue syndrome whose core pathophysiology is closely associated with reduced mixed venous oxygen tension caused by decreased cardiac output. This hypoxic state ultimately leads to mild to moderate hypoxemia in patients [27]. Previous studies have demonstrated that persistent hypoxia activates HIF‐1α, a key regulator of pulmonary hypoxia adaptation, resulting in pulmonary hypertension [28, 29]. Similarly, in our research, compared with the control groups, both the hypoxia‐induced PASMC and MCT‐induced PAH groups presented significantly higher HIF‐1α expression levels. Chronic hypoxia, a major trigger of PAH, induces pathological changes in pulmonary vascular remodelling, including pulmonary arteriole myxification, excessive proliferation of pulmonary arterial smooth muscle cells (PASMCs), abnormal apoptosis and activation of inflammatory responses and oxidative stress [30]. Deng et al. reported that calpain‐1 and HIF‐1α expression was increased in the lung tissues of mice and PASMCs subjected to hypoxia. Following calpain‐1 downregulation, hypoxia‐induced abnormal proliferation and migration of PASMCs were dampened and the expression of HIF‐1α was suppressed. Thus, targeting calpain‐1 reverses hypoxia‐induced pulmonary vascular remodelling and fibrosis, activating HIF‐1α signalling [31]. Here, we also confirmed that the HIF‐1α inhibitor LW6 can partly reverse hypoxia‐induced dysfunction in PASMCs.

As a major regulator of the hypoxia response, HIF‐1α can control the expression of miRNAs under hypoxia [32, 33]. Here, we revealed that both HIF‐1α and miR‐663b expression was increased in the PAH model. To investigate the mechanism of the HIF‐1α‐miR‐663b axis in PAH development, we conducted experiments in which we used miR‐663b inhibitors, HIF‐1α overexpression and the HIF‐1α inhibitor LW6 to investigate their effects on hypoxic PASMCs. The results demonstrated that HIF‐1α repressed miR‐663b expression under both normal and hypoxic conditions. miR‐663b inhibition improved HIF‐1α‐induced PASMC migration and inflammation and reduced the expression of PANoptosis‐associated proteins. These findings suggest a direct interaction between HIF‐1α and miR‐663b in PAH.

PANoptosis is a newly discovered form of programmed cell death that integrates three classical pathways: pyroptosis, apoptosis and necroptosis [34]. As the first confirmed form of programmed cell death, apoptosis was first demonstrated in 1996 when researchers observed endothelial apoptosis in the early stages of MCT‐induced PAH [35]. Apoptosis, a highly conserved form of programmed cell death, is mediated by cascade activation of the caspase family. The classical pathways include the mitochondrial pathway (activation of Bax/Bak leads to cytochrome C release, triggering caspase‐9/‐3) and the death receptor pathway (activation of Fas/TNF‐R triggers caspase‐8/‐3) [36, 37]. Pyroptosis is an inflammation‐related form of programmed cell death that is dependent on the activation of the GSDMD protein. After inflammasomes (such as NLRP3) recognise damage‐associated molecular patterns (DAMPs, such as HMGB1 and ATP), they activate caspase‐1/4/5/11. These activated caspases then GSDMD to generate N‐terminal fragments, which form pores in the cell membrane. This pore formation leads to a cellular osmotic imbalance and cell swelling and rupture. Moreover, it promotes the extracellular release of proinflammatory cytokines, including IL‐1β and IL‐18, thereby forming a positive feedback loop between cell death and inflammation [38]. This mechanism has been verified in PAH models. Shi et al. [39] reported increased protein expression levels of IL‐18, caspase‐1 and HMGB1 in the lung tissues of PAH model rats induced by MCT, which facilitated pyroptosis. Necroptosis, also known as programmed necrosis, relies on a mechanism in which the RIPK1‐RIPK3 complex forms a necrosome. This necrosome activates the phosphorylation of the MLKL protein, which then translocates to the cell membrane. This translocation results in increased membrane permeability, organelle swelling and LDH release, thereby triggering an intense inflammatory response [40]. Therefore, inhibiting proliferation, apoptosis, necroptosis and anti‐inflammatory and antioxidant stress in PASMCs is an effective strategy for treating PAH. Research by He et al. [41] revealed that PRDC suppresses apoptosis in distal PASMCs by downregulating caspase 3, caspase 9 and Bax expression. Zheng et al. [42] demonstrated that ZBP1 acts as a key ‘switch’ in PANoptosis, interacting with RIPK3 and other related molecules to form a ZBP1‐PANoptosome complex that induces cell death. Consistent with the findings of previous studies, we observed elevated expression of ZBP1, the pyroptosis executor GSDMD, the apoptosis executor caspase‐3/‐8 and the necrosis executor RIPK1/MLKL in both PAH rat models and PASMCs. Furthermore, miR‐663b expression was elevated in both in vivo and in vitro PAH models, suggesting that miR‐663b may play a central regulatory role in hypoxic pulmonary vascular cell injury by activating the pyroptosis, apoptosis and necrosis pathways.

As a heterotrimeric complex, AMPK is composed of an α catalytic subunit, a β regulatory subunit and a γ regulatory subunit. Its activity depends on the phosphorylation of the conserved Thr172 residue at the N‐terminus of the α‐subunit [43]. In mammals, LKB1 and calcium/calmodulin‐dependent protein kinase β (CaMKKβ) are the main upstream kinases that phosphorylate Thr172 and they activate AMPK through a cascade reaction [44]. In addition to classical upstream kinases, AMPK can also be indirectly activated by a variety of drugs, such as metformin, thiazolidinediones and polyphenolic compounds. These drugs trigger AMPK activation by altering the cellular energy status [45]. In patients with PAH, metformin treatment significantly improved right ventricular fractional area changes, possibly by modulating lipid and glucose metabolism [46]. Therefore, activating the AMPK pathway is a promising strategy for treating PAH. Studies have reported that miRNAs are vital mediators of the AMPK pathway. The inhibition of miRNAs can increase the activity of AMPK [47, 48, 49]. Notably, activated AMPK can effectively prevent the remodelling of the extracellular matrix in pulmonary arteries [50]. In a PAH pathological model, MCT‐induced pulmonary tissue in PAH rats presented significantly reduced AMPK phosphorylation levels [49]. Our research revealed that inhibiting miR‐663b promotes AMPK phosphorylation and exacerbates PASMC dysfunction.

**FIGURE 9:**
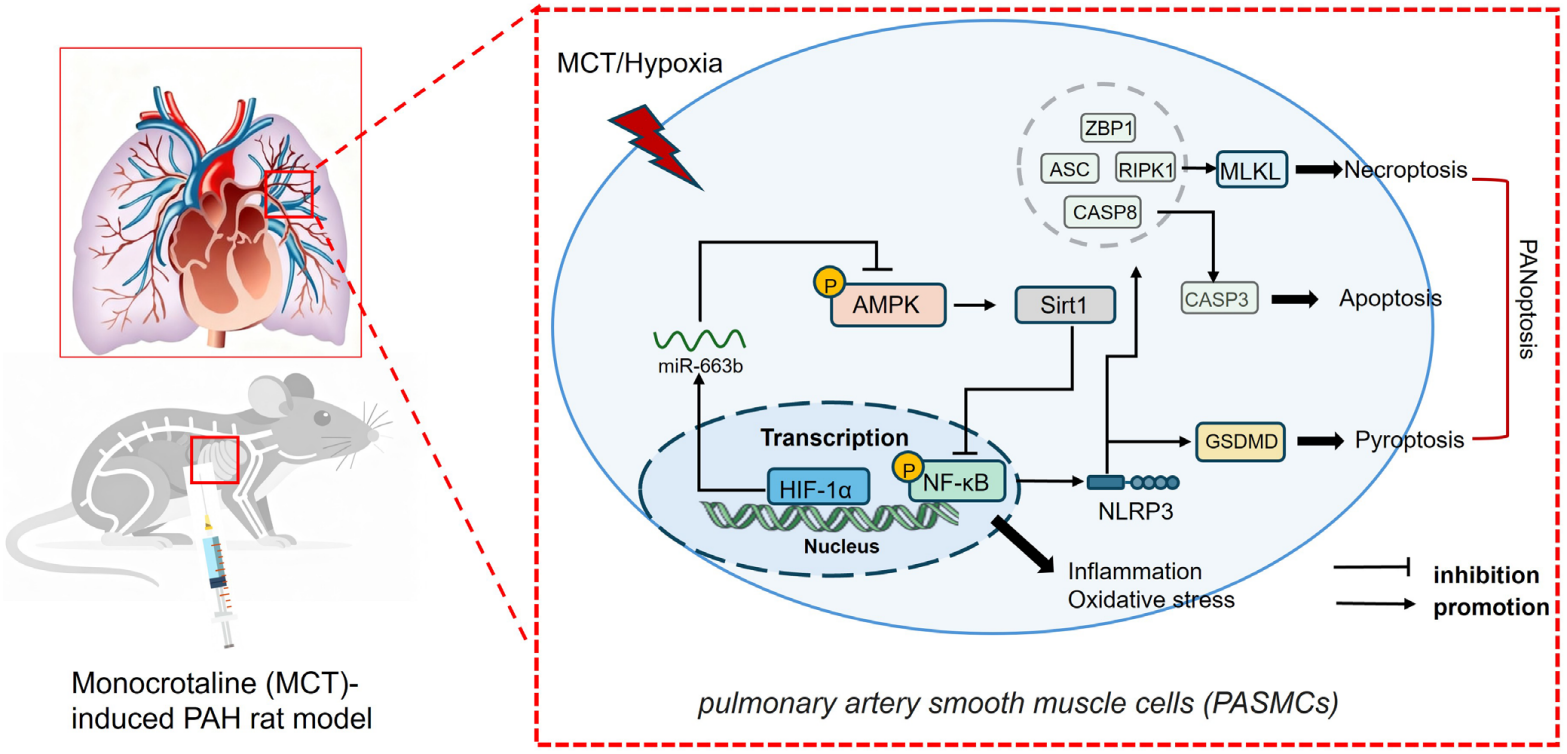
Mechanistic diagram of the HIF‐1α/miR‐663b/AMPK/Sirt1 axis in PAH and dysfunction of PASMCs. Hypoxia triggers HIF‐1α nuclear translocation and activates miR‐663b transcription. miR‐663b reduces Sirt1 expression by inhibiting AMPK phosphorylation, thereby weakening its inhibitory effect on NF‐κB nuclear translocation. Activated NF‐κB subsequently activates the NLRP3 inflammasome, which mediates pyroptosis, apoptosis and necroptosis through GSDMD, Casp3 and MLKL, respectively.

As a downstream target gene of AMPK, Sirt1 shares metabolic sensing roles with AMPK. The deacetylase activity of Sirt1 is highly dependent on the intracellular NAD^+^ concentration, whereas AMPK indirectly elevates NAD^+^ levels by regulating the NAD^+^/NADH redox state, thereby activating Sirt1 [21, 51]. Resveratrol, a Sirt1 activator, can repress the proliferation of PASMCs and prevent right ventricular remodelling, thus improving PAH through various signalling pathways [52]. A previous study revealed that Sirt1 activation exerts dual inhibitory effects on the NF‐κB inflammatory pathway, either by blocking NF‐κB nuclear translocation to suppress the transcription of proinflammatory cytokines such as TNF‐α and IL‐6 or by directly inhibiting NF‐κB to prevent downstream NLRP3 inflammasome assembly [53]. NF‐κB activation triggers the formation of the NLRP3/ASC/caspase‐1 complex, promoting the maturation and secretion of pro‐IL‐1β and pro‐IL‐18 and ultimately inducing caspase‐1‐dependent pyroptosis [54]. Consistent with previous studies, we observed that inhibiting miR‐663b increased Sirt1 protein levels while downregulating the expression of NLRP3, ASC and cleaved caspase‐1. Both the use of an AMPK inhibitor (compound C) and a SIRT1 inhibitor (Ex‐527) increased the viability, proliferation and migration of PASMCs, as well as the occurrence of apoptosis and necrosis, thereby antagonising the protective effects of the miR‐663b inhibitor on hypoxic PASMCs. The evidence indicates that inhibiting miR‐663b may alleviate hypoxia‐induced PAH by activating AMPK/Sirt1 signalling, which regulates apoptosis, pyroptosis and necrosis (Figure 9).

In conclusion, our study demonstrated that inhibiting the HIF‐1α‐mediated miR‐663b axis is a novel therapeutic strategy for PAH through the reduction of proliferation, inflammation and PANoptosis in hypoxia‐induced PASMCs through the activation of the AMPK/Sirt1 pathway. These findings suggest that suppressing the HIF‐1α‐miR‐663b axis may serve as a promising therapeutic strategy and a reliable biomarker for preventing PAH.

## Author Contributions

**Honghong Ma:** conceptualization, methodology, validation, formal analysis, writing – review and editing, writing – original draft. **Bing Zhuan:** conceptualization, funding acquisition, writing – original draft, writing – review and editing, visualization, project administration, formal analysis. **Yang Yu:** investigation, validation, software, methodology. **Qun Yuan:** conceptualization, validation, methodology, formal analysis, data curation. **Le Shen:** conceptualization, investigation, writing – original draft, writing – review and editing, visualization, formal analysis, software, supervision, data curation. **Yan Xu:** conceptualization, investigation, visualization, validation. **Xia Zhao:** resources, visualization, validation, methodology.

## Funding

This research was funded by the National Natural Science Foundation of China (No. 82160017), Ningxia Natural Science Foundation (Nos. 2024AAC03461 and 2025AAC030407) and Natural Science Foundation of Ningxia Province.

## Ethics Statement

This study was approved by the Ethics Committee of the People's Hospital of Ningxia Hui Autonomous Region (2021‐GZR‐054).

## Conflicts of Interest

The authors declare no conflicts of interest.

## Data Availability

The data that support the findings of this study are available on request from the corresponding author. The data are not publicly available due to privacy or ethical restrictions.
